# A Role for the Krebs Cycle Intermediate Citrate in Metabolic Reprogramming in Innate Immunity and Inflammation

**DOI:** 10.3389/fimmu.2018.00141

**Published:** 2018-02-05

**Authors:** Niamh C. Williams, Luke A. J. O’Neill

**Affiliations:** ^1^School of Biochemistry and Immunology, Trinity Biomedical Sciences Institute, Trinity College Dublin, Dublin, Ireland

**Keywords:** immunometabolism, citrate, ATP-citrate lyase, itaconate, acetylation, metabolism, macrophages, Krebs cycle

## Abstract

Metabolism in immune cells is no longer thought of as merely a process for adenosine triphosphate (ATP) production, biosynthesis, and catabolism. The reprogramming of metabolic pathways upon activation is also for the production of metabolites that can act as immune signaling molecules. Activated dendritic cells (DCs) and macrophages have an altered Krebs cycle, one consequence of which is the accumulation of both citrate and succinate. Citrate is exported from the mitochondria *via* the mitochondrial citrate- carrier. Cytosolic metabolism of citrate to acetyl-coenzyme A (acetyl-CoA) is important for both fatty-acid synthesis and protein acetylation, both of which have been linked to macrophage and DC activation. Citrate-derived itaconate has a direct antibacterial effect and also has been shown to act as an anti-inflammatory agent, inhibiting succinate dehydrogenase. These findings identify citrate as an important metabolite for macrophage and DC effector function.

## Metabolic Reprogramming in Macrophages and Dendritic Cells (DCs)

The innate immune system is the first line of defense against infection. Cells of the innate immune system have a range of germline encoded receptors, pathogen recognition receptors that allow for the recognition of pathogen-associated molecular patterns, and danger-associated molecular patterns from damaged cells or tissues (1, 2). Macrophages and DCs play an important role in the initiation and resolution of the immune response. Both can produce inflammatory mediators, phagocytose pathogens and release chemokines to recruit other immune cells to the site of infection (3). DCs are also important in the activation of naive T cells as they can present antigen to the T cell initiating an adaptive immune response (4).

The dual role played by macrophages in initiation and resolution of inflammation requires cells to adopt different processes. In macrophages, this can be broadly described in terms of M1, lipopolysaccharide (LPS)- or classically activated macrophages, and M2, IL-4-activated macrophages. M1 macrophages are more pro-inflammatory and will produce inflammatory mediators, such as nitric oxide (NO) and reactive oxygen species (ROS) (3, 5). M2 macrophages are important in helminth infection and the resolution of inflammation, secreting growth factors to aid in tissue repair and regeneration and cytokines such as IL-10 that can dampen the immune response (3, 6).

Both macrophages and DCs must be able to switch rapidly from a resting to an activated state. A hallmark of immune cell activation is a change in their metabolism. M1 macrophages upregulate glycolysis and the pentose phosphate pathway (PPP) while the Krebs cycle is broken at two points and the fatty acid oxidation (FAO) and oxidative phosphorylation (OXPHOS) are downregulated (5). Toll-like receptor (TLR)-activated DCs also have increased aerobic glycolysis and decreased OXPHOS and FAO (7). This inhibition of mitochondrial respiration in murine DCs is due to NO and long-term activation of glycolysis in activated DCs serves to produce adenosine triphosphate (ATP) to compensate for the collapse in mitochondrial function, maintain the mitochondrial membrane potential (Δψ_M_) and prevent cell death (8). The high rate of glycolysis is similar to that seen in tumor cells (3). Murine M2 macrophages also upregulate glycolysis, but the Krebs cycle is intact and OXPHOS is functioning (5). A general theme exists among immune cells where a reliance on aerobic glycolysis is important for cells, such as M1 macrophages and DCs, whereas immunomodulatory cells, such as M2 macrophages and regulatory T cells (Tregs), make use of OXPHOS (9).

Recent work has shed light on several of the key determinants of metabolic reprogramming in M1 macrophages and DCs. The upregulation of inducible nitric oxide synthase (iNOS) and resulting generation of NO causes inhibition of mitochondrial respiration in murine cells (10–12). Hypoxia-inducible factor-1α (HIF1α) can be induced under normoxic conditions in immune cells and this is crucial for upregulation of glycolysis (13). HIF-target genes included those encoding for glycolytic enzymes, the glucose transporter GLUT1, and lactate dehydrogenase (LDH), as well as inflammatory factors such as interleukin-1β (IL1β) (14–16). LPS-treatment of macrophages or DCs activates the mammalian target of rapamycin (mTOR), a central regulator of metabolism, which in turn boosts expression and activity of HIF1α (17). Finally AMP-activated protein kinase (AMPK) is inhibited by LPS treatment (18). AMPK senses the energy status of a cell, and when that is low this inhibits anabolic pathways and drives catabolic ones such as FAO, while also inhibiting mTOR and nuclear factor-κB (NF-κB) signaling. Its inhibition by LPS allows changes necessary for a pro-inflammatory response to occur. Several processes that are upregulated in M1 macrophages are downregulated in M2 macrophages. In mouse macrophages, iNOS expression is decreased and arginase-1 (ARG1) is highly upregulated, and so arginine is preferentially metabolized to proline and polyamines (19). mTOR is inhibited by activation of upstream repressors TSC1 and TSC2 while AMPK activity is high (18, 20).

It is possible that the upregulation and reliance on aerobic glycolysis is in part due to the rate of response required of these cells for an effective immune system. Glycolysis, though less efficient at producing ATP, is able to do so more rapidly than OXPHOS. However, there is evidence that upregulation of metabolic pathways is more nuanced than that. Metabolic changes are important not only in terms of generating biosynthetic precursors and for ATP production, but it also has emerged that metabolites themselves can act as signaling molecules and affect important inflammatory pathways (21, 22). Of particular interest are metabolites of the Krebs cycle. The oxidation of succinate by succinate dehydrogenase (SDH) has been shown to be of importance in the classical activation of macrophages (23). This leads to reverse electron transport (RET) in complex I of the electron transport chain (ETC) driving the production of ROS, which in turn leads to activation of HIF1α. Increased levels of cytosolic succinate can inhibit the prolyl hydroxylase domain enzymes *via* product inhibition, also potentiating HIF1α stabilization (24). This prevents the hydroxylation of proline residues on HIF1α, and so it is not ubquitinated and targeted for proteasomal degradation (25–28). Instead, it can heterodimerize with its binding partner the aryl hydrocarbon nuclear translocator (ARNT/HIF-1β). The HIF-1 complex can translocate to the nucleus and bind hypoxia response elements in the promoters of HIF target genes (29). HIF also represses mitochondrial function through upregulation of pyruvate dehydrogenase kinase 1 (PDK1) (30). PDK1 phosphorylates and inhibits pyruvate dehydrogenase (PDH) and so pyruvate cannot be converted into acetyl-CoA in order to enter the mitochondria and feed the Krebs cycle (31).

The fragmented Krebs cycle in macrophages is not only due to the break after succinate. A second breakpoint, at isocitrate dehydrogenase (IDH), allows for the withdrawal of citrate from the cycle. This proves not only to be important for lipid biosynthesis in macrophages and DCs, but also for the production of both pro- and anti-inflammatory mediators (32, 33). Glycolysis is rapidly upregulated in LPS-activated DCs for the production of citrate. This is necessary for the upregulation of fatty acid synthesis to allow for membrane expansion which is crucial for antigen presentation (34). Once exported to the cytosol citrate can be broken down to provide a source of acetyl-CoA for acetylation of both histone and non-histone proteins (35). Citrate metabolism provides a connection between carbohydrate metabolism, fatty acid metabolism, and epigenetic reprogramming and so changes in flux through this pathway may have wide ranging effects. This review will describe the role of citrate in innate immune cell function.

## Citrate Provides a Bridge Between Carbohydrate and Fatty Acid Metabolism

**Figure 1:**
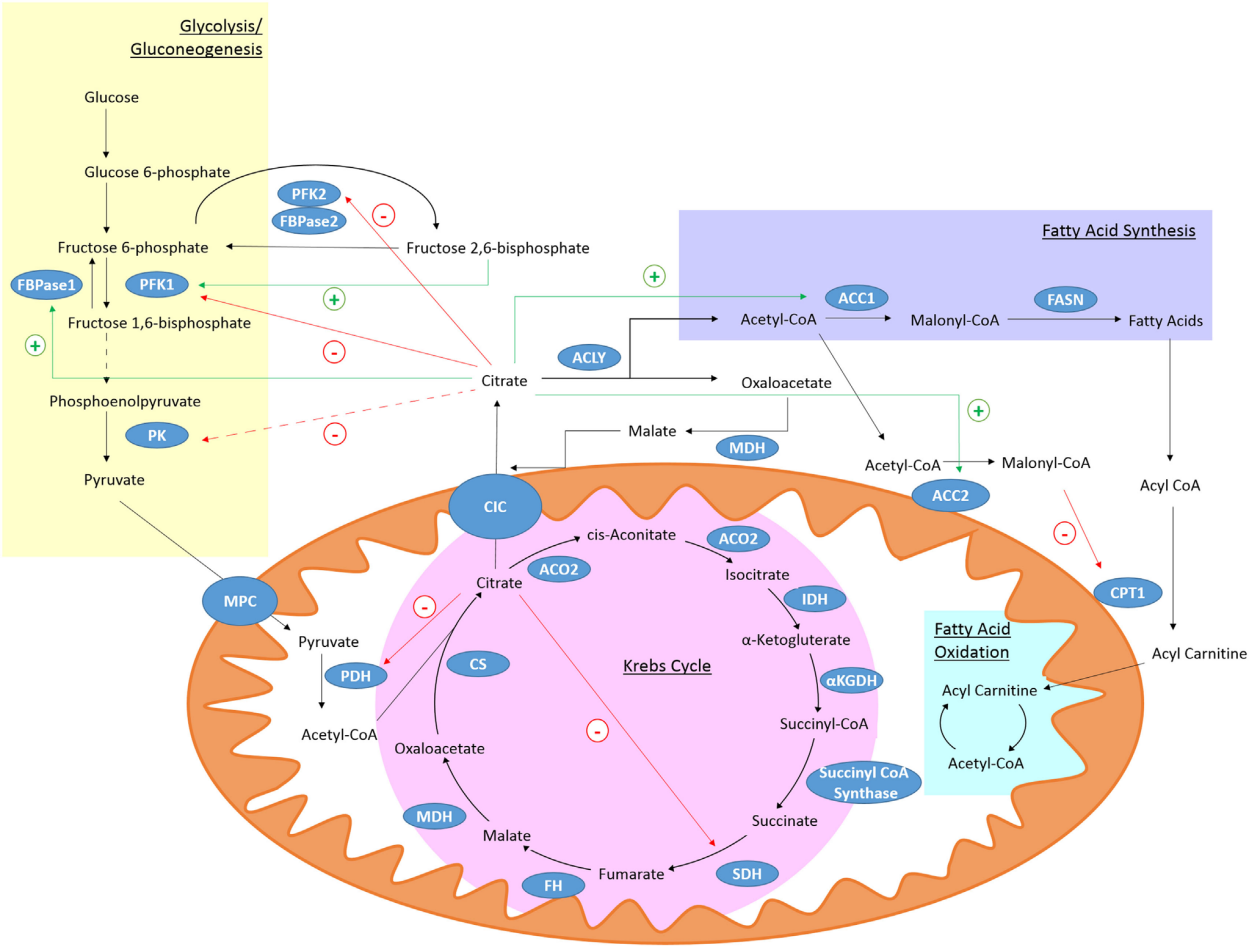
Citrate metabolism. Citrate is produced in the Krebs cycle from oxaloacetate and acetyl-CoA by citrate synthase (CS). It can be exported from the mitochondria through citrate carrier (CIC). Cytosolic citrate is broken down by ACLY to oxaloacetate and acetyl-CoA. Acetyl-CoA can be used as a substrate for fatty acid synthesis. High levels of cytosolic citrate can directly inhibit the glycolytic enzymes PFK1 and PFK2. PFK1 is also indirectly inhibited by decreased levels of fructose 2,6-bisphosphate and pyruvate kinase (PK) is indirectly inhibited (broken line) due to reduced levels of its activator, fructose-1,6-bisphosphate. Mitochondrial citrate can inhibit pyruvate dehydrogenase (PDH) and succinate dehydrogenase (SDH). Citrate-derived malonyl-CoA can block fatty acid oxidation (FAO) by inhibiting carnitine palmitoyltransferase 1 (CPT1). Citrate can activate the gluconeogenic enzyme fructose 1,6-bisphosphatase (FBPase1) and ACC, and so stimulate fatty acid synthesis.

Citrate is produced in the Krebs cycle (also known as the citric acid cycle or TCA cycle) from the aldol condensation of oxaloacetate, the end product of a previous turn of the cycle, and acetyl-CoA (Figure 1) (36). Acetyl-CoA may be derived from glucose *via* the glycolytic pathway, entering the mitochondria as pyruvate or from fatty acids that have undergone β-oxidation (36). In the Krebs cycle, citrate is converted into isocitrate *via* cis-aconitate by aconitase (36). IDH will then convert isocitrate to α-ketogluterate (αKG) in a decarboxylation reaction (36). The Krebs cycle continues and provides a major source of cellular ATP and also reducing equivalents that feed the electron transfer chain (36).

The mitochondrial citrate carrier (CIC), also known as solute carrier family 25 member 1 (Slc25a1), can export citrate from the mitochondria in exchange for malate (37). Once in the cytosol citrate is broken down by ATP-Citrate lyase (ACLY) into acetyl-CoA and oxaloacetate (37). Oxaloacetate can be converted to malate by malate dehydrogenase (MDH) which can re-enter the mitochondria through CIC (37). Acetyl-CoA is further processed into malonyl-coenzyme A (malonyl-CoA) by acetyl-coA carboxylase (ACC) (38). Malonyl-CoA can be incorporated into cholesterol or fatty acids (38). The fatty acids are incorporated into phospholipids. Malonyl-CoA can also limit the β-oxidation of fatty acids as high levels can inhibit carnitine palmitoyltransferase 1 (CPT1) (39). Two isoforms of ACC exist, ACC1 and ACC2 (40). ACC2 is associated with the outer mitochondrial membrane and so can control the concentration of malonyl-CoA near CPT1 and regulate its activity (38). Acetyl-CoA can also be a substrate for protein and histone acetylation and so can have a wide ranging role in many cellular processes (41).

Citrate itself is known to inhibit several key glycolytic enzymes as part of a negative feedback loop. Phosphofructokinase (PFK) 1 and 2 are directly inhibited by citrate while pyruvate kinase (PK) is indirectly inhibited as citrate decreases levels of fructose-1,6-bisphosphate, which is a PK activator (42). PDH (43) and SDH (44), and therefore the Krebs cycle, can also be inhibited by high citrate levels. While citrate inhibits pathways producing ATP, it stimulates those that consume it. Citrate can allosterically activate ACC (45) and the gluconeogenic enzyme fructose-1,6-bisphosphatase (46). With citrate occupying a position that links many metabolic and cellular processes, it is not surprising that the metabolism of citrate may be of importance in the immune response.

## Citrate as an Inflammatory Signal

**Figure 2:**
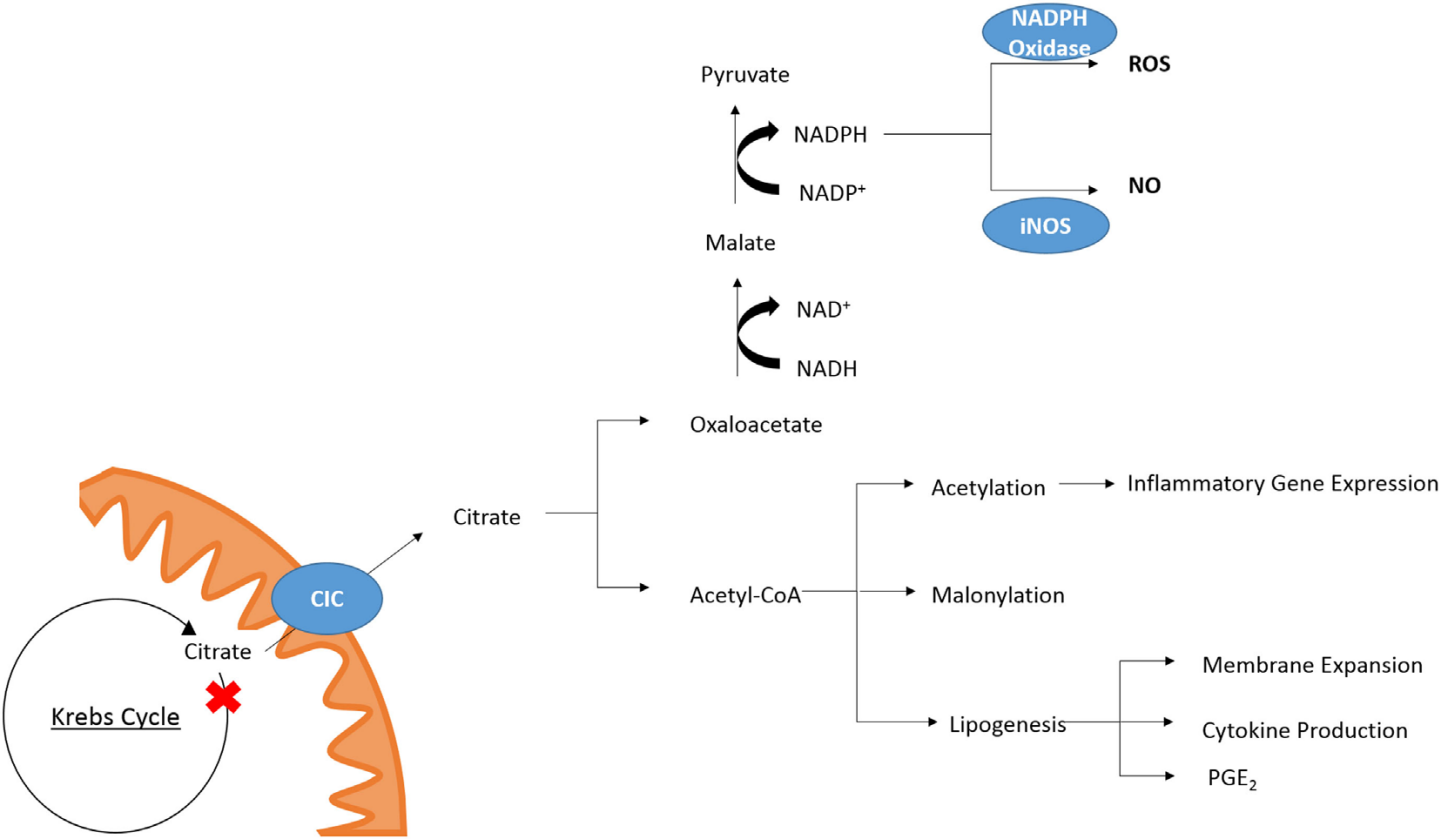
Citrate in inflammation. Cytosolic citrate is broken down into acetyl-CoA and oxaloacetate. Further processing of oxaloacetate can provide a source of NADPH which is required by NAPDH oxidase and iNOS for the production of ROS and NO, respectively. Acetyl-CoA is required for acetylation of proteins and can be converted to malonyl-CoA for lysine-malonylation. Citrate-derived lipid synthesis has been linked to membrane expansion necessary for DC activation and cytokine production. In macrophages, the production of PGE_2_ has been linked to citrate metabolism.

While the role of other metabolites as inflammatory signals has been well discussed (21, 22) citrate also plays a role in key inflammatory pathways (Figure 2). In M1 macrophages, there is an increased isocitrate:αKG ratio and transcriptional downregulation of *Idh1* (47). This break was also seen in DCs (34). With increased glycolytic flux in both activated DCs and macrophages and a break in the Krebs cycle, pyruvate derived from glucose feeds into Krebs cycle but cannot continue past citrate/isocitrate. An increase in the levels of citrate is detected in both mouse (LPS-stimulated) and human [tumour necrosis factor α (TNFα)- or interferon-γ (IFNγ)-stimulated] macrophages (24, 48). This coincides with upregulation of CIC and ACLY, both of which occur in an NF-κB-dependent manner where LPS or TNFα is used to activate the cells, or IFNγ can also induce CIC and ACLY *via* STAT1 (32, 49). The export and breakdown of mitochondrial citrate has been linked to the production of several important pro-inflammatory mediators in macrophages, namely NO, ROS, and prostaglandin E_2_ (PGE_2_) production in human macrophages (32, 48, 49). Inhibition of CIC activity or its genetic silencing with siRNA leads to a marked reduction in NO, ROS, and PGE_2_ production in LPS and cytokine-stimulated macrophages. Infantino et al. suggest that the decrease in PGE_2_ production is due to a decreased availability of precursors for PGE_2_ synthesis as adding exogenous acetate rescues the effect of CIC inhibition on PGE_2_ productions. Acetate can be converted to acetyl-CoA by acetyl-CoA synthase (ACSS) (50). Inhibition of fatty acid synthase (FASN) with C75 in DCs also reduced LPS-induced PGE_2_ (34). Endogenous PGE_2_ is essential for the production of LPS-induced pro-IL1β (51). This implies that citrate may be critical to IL1β production.

While Infantino et al. speculated that the effect of CIC inhibition on the production of NO and ROS was due to decreased production of NADPH by the breakdown of citrate-derived oxaloacetate to pyruvate by malic enzyme, a follow-up study reports that the NADP^+^/NAPDH ratio is unchanged in cytokine-activated macrophages treated with the CIC inhibitor 4-chloro-3-[(3-nitrophenyl)amino]sulfonyl benzoic acid (CNSB) versus untreated when they are in a glucose rich environment (52). When the same experiment was carried out in glucose-deprived conditions, there was a marked increase in the NADP^+^/NADPH ratio in activated, CNSB-treated macrophages compared to control cells. Citrate export from the mitochondria can provide a source of NADPH *via* two routes. The first is from oxaloacetate as mentioned, and the second is *via* the cytosolic, NADP^+^-dependent IDH (IDH1). IDH1 is known to be suppressed in cells activated by a pro-inflammatory stimuli (53), however, in glucose-deprived conditions the authors show *IDH1* mRNA expression is increased in activated macrophages (52). While this could provide a mechanism for the decreased production of NO and ROS in glucose-deprived cells other processes such as the PPP may be compensating for decreased production of NADPH in macrophages treated with a CIC inhibitor but grown in a glucose-rich environment.

The processing of citrate by ACLY has also been suggested as an anti-cancer target as ACLY has been found to be overexpressed in several cancer types (54). Inhibition of ACLY with siRNA or pharmacologically induces cell death (55–58). One study has suggested that the anti-cancer effect of ACLY inhibition is directly due to its role in lipid synthesis (55). In several cancer cell lines, depletion of ACLY was observed to induce apoptosis, accompanied by increased levels of ROS. This increase in ROS was suggested to initiate cell death by activating AMPK (59). Another study has suggested that enzymatic activity alone is not enough to explain the initiation of growth arrest in tumor cells, and has shown that ACLY may be able to directly interact with AMPK (60). While ACLY has not been linked to the production of ROS in immune cells, an exact mechanism for the role of citrate metabolism in macrophages has not been defined and it would be an interesting avenue of investigation.

Similarly, citrate has been shown to be important for DC activation. Mitochondrial respiration in blood monocyte-derived DCs will begin to collapse in TLR-activated DCs 6 h after treatment, and by 24 h, there will be no detectable consumption of oxygen by the mitochondria (8). However, before 6 h the mitochondria are still functioning. Up until this time, the increase in glycolysis is important for DC activation but the increase does not fuel a greater rate of OXPHOS, inhibiting ATP synthase with oligomycin has no effect on early DC activation. Instead citrate is withdrawn from the Krebs cycle and used to support *de novo* fatty acid synthesis (34). Inhibition of ACC or FASN, with TOFA and C75, respectively, or blocking expression of *Slc25a1* by retroviral introduction of shRNA diminished the early activation of DCs differentiated from bone marrow in the presence of the growth factor GM-CSF (GM-DCs). Production of fatty acids is required for membrane synthesis for the expansion of both the endoplasmic reticulum and Golgi. Their expansion allows for increased synthesis and secretion of various proteins important following TLR-activation. The PPP is also an important process in early DC activation, as it creates the reducing equivalent, NADPH, required not only for nucleotide synthesis and redox balance but also as a cofactor for lipogenesis. While the long-term commitment to glycolysis and collapse in mitochondrial respiration seen in activated DCs is dependent on a phosphatidylinositide-3-kinase (PI3K)/Akt pathway, the rapid upregulation of glycolysis was due to TANK-binding kinase 1 (TBK1)- and IκB Kinase ε (IKKε)-dependent activation of Akt. The PI3K/Akt pathway lead to production of NO, and inhibition of OXPHOS, forcing the cells to rely on increased glycolysis for activation and survival but the early TBK1-IKKε/Akt pathway increased glycolysis by promoting the association of hexokinase 2 (HK2) with voltage-dependant anion channels on the outer mitochondrial membrane.

While the same breakpoints in the Krebs cycle have not been described in activated natural killer cells (NK cells), the citrate–malate shuttle has recently been shown to be of importance in the metabolic reprogramming that occurs following NK cell activation (61). The citrate–malate shuttle refers to the export of citrate into the cytosol *via* CIC, and its breakdown by ACLY and malate dehydrogenase 1 (MDH1) yielding cytosolic malate which CIC exchanges for citrate. Assmann et al. show that increased expression of *Slc25a1* and *ACLY* mRNA in activated NK cells is dependent on sterol regulatory element-binding protein (SREBP) activity, which is consistent with reports in other cell types including macrophages (62–66). Activated NK cells have increased glycolysis and OXPHOS which is crucial for their activation and growth. Pharmacological inhibition of SREBP activation or ACLY activity (therefore, inhibiting the citrate–malate shuttle) reduced cytokine-induced granzyme B expression and IFN-γ production. Similar results were seen with genetic inhibition of SREBP using SCAP^−/−^ mice. This effect is independent of lipid and cholesterol synthesis downstream of ACLY as inhibition of ACC or FASN with TOFA or C75, respectively, does not affect NK cell activation. The authors speculate that the citrate–malate shuttle serves to convert cytosolic NADH to mitochondrial NADH, therefore, fueling OXPHOS and mitochondrial ATP synthesis while also replenishing the cytosolic pool of NAD^+^, which is an important cofactor for glyceraldehyde 3-phosphate dehydrogenase (GAPDH). Inhibition of the malate–aspartate shuttle, which would be more commonly thought of as a means of replenishing mitochondrial NADH, did not affect OXPHOS in activated NK cells and neither did inhibition of the Krebs cycle enzyme SDH with dimethyl-malonate. This suggests that in NK cells elevated OXPHOS is maintained by the citrate–malate shuttle. While these studies show the cytosolic processing of citrate to be generally a pro-inflammatory event, citrate itself has been shown to inhibit the HIF asparaginyl hydroxylase [factor inhibiting HIF (FIH)] (67). FIH hydroxylates asparagine residues on HIF1α, preventing it from interacting with transcriptional coactivators such as CRE binding protein/p300 (67, 68). Increased flux through CIC and the cytosolic processing of citrate has, therefore, been shown to be of importance in the activation of macrophages, DCs, and NK cells.

## Histone and Post-Translational Modifications by Citrate-Derived Acetylation

Acetyl-CoA is not only a substrate for *de novo* lipogenesis, it also is an important cofactor for the acetylation of histones and non-histone proteins (69). Acetylation can be co-translational, effecting the α-amino group of a protein’s N-terminal residue, or post-translational which concerns the ε-amino group of lysine residues (69, 70). Lysine acetylation is reversible and so provides a very useful mechanism for the regulation of gene expression and general protein function (69). Acetyl-CoA cannot travel across cell membranes, and so to exert its effects it must be generated in different cellular compartments (70). In the mitochondria, acetyl-CoA is present due to the β-oxidation of fatty acids or is generated from pyruvate by the PDH complex (41). In the cytosol, acetyl-CoA can be derived from citrate as previously discussed or from acetate by ACSS (71) or the degradation or N-acetylaspartate (NAA) by aspartoacylase (72). The generation of acetyl-CoA from NAA is more commonly associated with processes in the brain and it has also been shown to be a source of nuclear and cytosolic acetyl-CoA in brown adipose tissue (73).

ATP-citrate lyase links metabolism to histone acetylation as it converts glucose-derived citrate to acetyl-CoA and it has been found to localize to both nucleus and cytoplasm (35). Citrate is small enough to diffuse across nuclear pores allowing for acetyl-CoA to be produced in either cellular compartment, and siRNA-mediated knockdown of ACLY reduced global histone acetylation (35). In adipocytes mRNA expression of HK2, PFK1, and lactate dehydrogenase A (LDHA) were all reduced when ACLY was silenced (35). Since ACLY is upregulated in lipopolysaccharide (LPS)-stimulated macrophages (49), it would be interesting to see where ACLY localized to and if there was a direct effect on the expression of glycolytic genes due to changes in histone acetylation. ACLY has been shown to control glucose to acetate switch. ACLY-deficient cells upregulate ACSS2 allowing for the production of acetyl-CoA from acetate, ensuring cell viability and providing substrates for both fatty acid synthesis and histone acetylation (74).

As previously discussed, in glucose-deprived conditions, increased flux through CIC can sustain NADPH levels in glucose-deprived activated macrophages (52). Acetylation of CIC increases in glucose-deprived growth conditions compared to media containing glucose. By reconstituting liposomes with mitochondrial extracts, it was shown that the acetylation of CIC causes an increase in V_max_ for citrate (52). In memory, CD8^+^ T cells GAPDH has been shown to be acetylated in an ACLY-dependent manner which increased its activity (75). Acetate is taken up and processed *via* the Krebs cycle to produce citrate. Citrate-derived acetyl-CoA was then used to acetylate GAPDH which may prevent it from binding IFNγ mRNA, and allowing its translation (76). Acetylated GAPDH had increased catalytic activity. While acetate can be used directly to generate acetyl-CoA, knockdown of the cytosolic ACSS (ACSS1) had no effect on IFNγ production, knockdown of ACLY, however, did decrease IFNγ production. It has also been shown that LDHA in T cells promotes the expression of IFNγ, independent of GAPDH regulation, as it ensures a high acetyl-CoA concentration produced *via* ACLY for histone acetylation (77). Though metabolic reprogramming differs in activated T cells compared to macrophages and DCs, this highlights the importance of the citrate pathway in control of both the metabolism of immune cells and their production of pro-inflammatory mediators, unlike in T cells. No work has yet been carried out to directly link citrate-derived acetylation in M1 macrophages or DCs, however, histone acetylation is important in macrophage activation and DC differentiation. IL-6 and IL-10 production are both regulated on histone and non-histone protein acetylation, respectively (78, 79). NF-κB activation is also dependent on acetylation of its RelA/p65 subunit (80) and a large number of enzymes involved in metabolic processes have been found to be acetylated in non-immune cells (78, 81). Therefore, it is likely that acetylation plays a role in the regulation of immune cell metabolism. Histone acetylation downstream of ACLY has been shown to be of importance in M2 macrophage activation (82). While STAT6 is the major regulator of IL4 induced genes a subset of genes important in the regulation of cellular proliferation and the production of chemokines are under additional control of an Akt–mTORC1 signaling pathway. Covarrubias et al. suggest a mechanism in which Akt regulates both protein levels and activity of ACLY to increase the acetyl-CoA pool for histone acetylation. They suggest that certain transcription factors and histone acetyltransferases, e.g., P300, are regulated by acetyl-CoA levels and that may link AKT/ACLY-dependent acetyl-CoA levels to specific gene induction.

Lysine-malonylation causes a net change in charge of the lysine residue from +1 to −1 and cause a change in mass of approximately 86 Da (83). Malonyl-CoA is the cofactor required (84). Malonyl-CoA can be produced in the cytosol from citrate-derived acetyl-CoA by ACC1/2 (38). The cytosolic pool of malonyl-CoA is regulated by both ACC and malonyl-CoA decarboxylase which catalyzes the reverse reaction from malonyl-CoA to acetyl-CoA (38). A malonyl-CoA pool also exists in the mitochondria, produced from acetyl-CoA by propionyl-CoA carboxylase or from malonate by acyl-CoA synthase family member 3 (ACSF3) (85). Lysine-malonylation has been shown to play a role in the regulation of mitochondrial function, FAO and glycolysis (86, 87). Notably histone malonylation does not occur at the N-terminal tail as happens with acetylation, suggesting that the regulatory role these two modification carry out may be very functionally different to acetylation (86). A large number of proteins involved in fatty acid metabolism are malonylated, including ACLY. However, no studies have yet been carried out regarding the functional consequence of lysine-malonylation in immune cells.

## Itaconate

**Figure 3:**
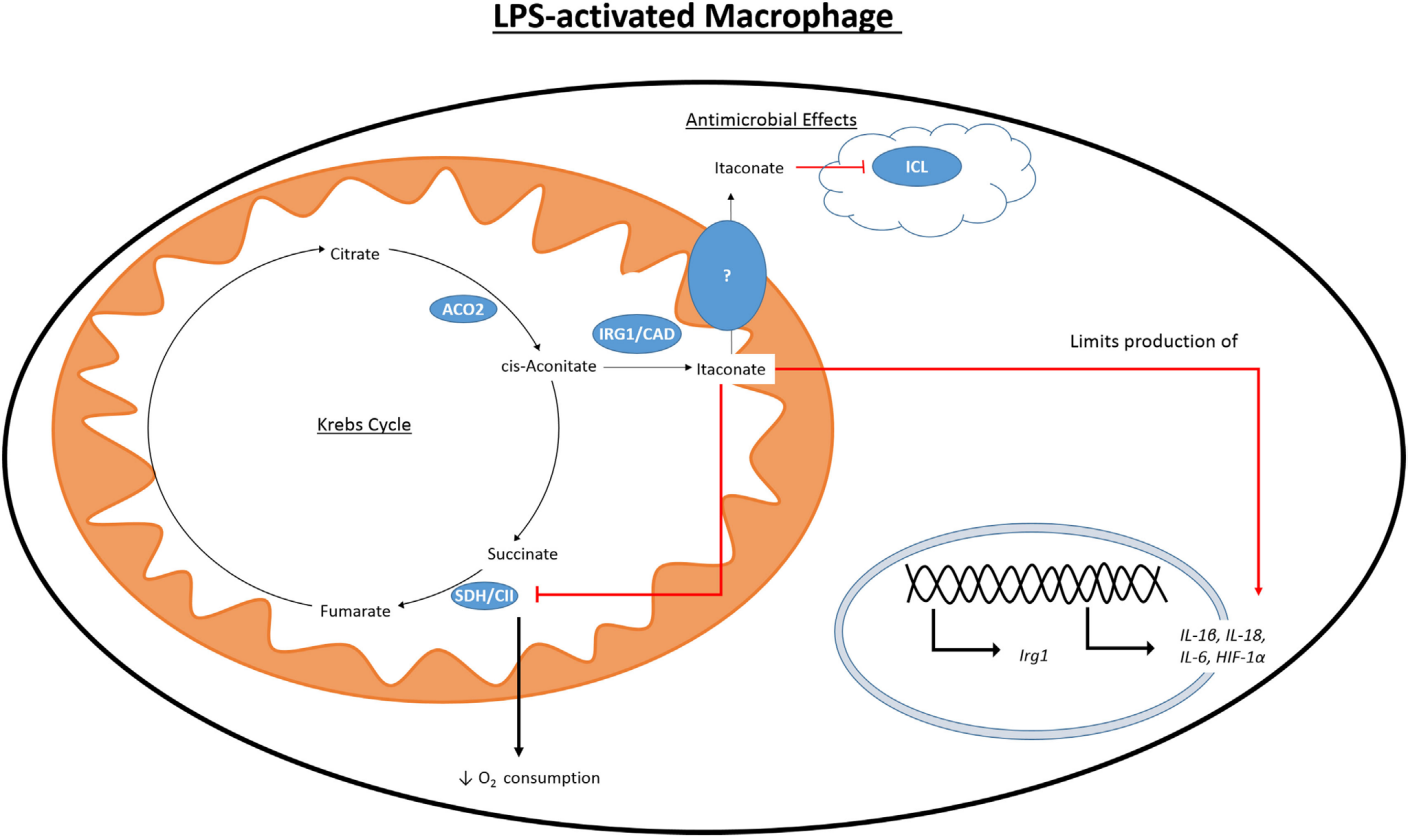
Citrate-derived itaconate. Citrate is converted to cis-aconitate by the Krebs cycle enzyme ACO2. Activation of macrophages with LPS causes induction of IRG1 which can produce itaconate by the decarboxylation of cis-aconitate. LPS also initiates the production of other pro-inflammatory mediators, such as HIF1 α, NO, and IL1β. Itaconate is toxic to microorganisms expressing ICL, a key component of the glycoxylate shunt in bacteria. Itaconate is also able to inhibit SDH and cause decreased production of NO, IL1β, IL18, and HIF1α.

While the accumulation of citrate caused by the IDH1 breakpoint in the TCA cycle can be used to fuel fatty acid synthesis and histone acetylation, another fate of this citrate is the production of itaconate (Figure 3). First identified in 1836 as a product of the distillation of citric acid, itaconate has recently become a focus of the field of immunometabolism due to its potential role as an anti-inflammatory modulator. Itaconate is derived from citrate produced in the Krebs cycle and, in M1 macrophages, is one of the most highly induced metabolites following LPS treatment (33). Itaconate has also been shown to accumulate in LPS-treated DCs (34). Citrate is acted on by the mitochondrial aconitase 2 (ACO2) to produce cis-aconitate. Cis-aconitate is decarboxlylated by cis-aconitate decarboxylase, also known as immune-responsive gene 1 (IRG1), to produce itaconate. Itaconate has long been used in an industrial setting and is produced on an industrial scale as a fermentation product of *Aspergillus terreus* for use in the creation of polymer formation (88).

In 1995, it was found that *immunoresponsive gene 1 (Irg1)* is highly upregulated in peritoneal macrophages following LPS stimulation (89), and has since been seen to be upregulated in the blood of human sepsis patients (90) and in the time during embryo implantation (91, 92). Despite lacking a sequence targeting it, there IRG1 has been found to associate with the mitochondria (93, 94). It was only in 2011 that itaconate was identified in multiple studies in an immune context and in 2013 that IRG1 and itaconate were connected (95). Itaconate was seen in the lungs of mice infected with *Mycobacterium tuberculosis* (MTB) and was not present in the lungs of control mice (96). In a separate study, itaconate was shown to be secreted by the macrophage cell line RAW264.7 following treatment with LPS (97). Michelucci et al. identified the role of IRG1 by siRNA-mediated silencing of *Irg1 (*officially renamed *Acod1* after its function was discovered) and performing a metabolic screen to elucidate which metabolites were affected. They further showed by isotope-labeling that itaconate was derived from citrate. Genetic silencing of *Irg1* causes macrophages to lose their bactericidal activity, which was due to decreased amounts of itaconate and the loss of its inhibitory effect on isocitrate lyase (ICL), a crucial enzyme of the glycoxylate shunt in bacteria (98). The glycoxylate shunt is a means for bacteria to survive in conditions of low glucose availability where acetate is the primary fuel source. Instead of the normal sequence of reaction in the Krebs cycle, the steps from αKG to fumarate are bypassed, and isocitrate is instead cleaved by ICL to glycoxylate and succinate in bacteria. Succinate enters the Krebs cycle and glycoxylate is then converted to malate by malate synthase. Malate can be processed to oxaloacetate by MDH as in the normal reactions of the Krebs cycle. Itaconate has been shown to inhibit growth of a number of ICL expressing microorganisms, including MTB, *S. enterica* and multidrug-resistant *Staphylococcus aureus* (MRSA) (95, 96, 99). Some bacteria are able to degrade itaconate, producing acetyl-CoA and pyruvate, due to the expression of genes that encode for itaconate-CoA transferase, itaconyl-CoA hydratase, and (S)-citramalyl-CoA ligase. Possession of these genes allows *Pseudomonas aeruginosa* and *Yersinia pestis* to survive in activated macrophages (100). There is a discussion as to the relevance of these studies due to differences in concentrations of itaconate used both in terms of the variety of concentrations used exogenously to inhibit bacterial growth and the range in reported intracellular concentrations (101, 102). It may be that intracellular itaconate is concentrated in vacuoles, and whole cell analysis will not adequately represent this, and that measuring the concentration of secreted itaconate in cell culture media does not determine what the local concentration would be.

While the effect of itaconate on bacterial survival has been well documented, more recent work has sought to elucidate the effect that a high intracellular concentration of itaconate has on the immune cells that produce it. Dimethyl itaconate (DMI) has been used in several studies as a cell permeable itaconate analog to boost the intracellular levels of itaconate. Pre-treatment of murine bone marrow-derived macrophages (BMDMs) with DMI prior to LPS stimulation reduced the reduced the expression of many pro-inflammatory genes including iNOS (33). DMI also impaired production of ROS, NO, IL1β, IL18, and IL6. While IL1β and IL18 mRNA expression was reduced by DMI it also caused a reduction in protein levels of both NLRP3 and ASC, indicating that inflammasome priming was also impaired. *Irg1*^−^*^/^*^−^ BMDMs do not produce itaconate and have increased production of NO, IL1β, IL18, and IL6 when compared to WT BMDMs. HIF1α protein levels were also increased in LPS-activated *Irg1*^−^*^/^*^−^ BMDMs while treatment with DMI inhibited its protein levels. Interestingly, *Irg1*^−^*^/^*^−^ BMDMs show an altered profile of Krebs cycle metabolites than WT cells. WT BMDMs have increased levels of succinate, fumarate, and malate following LPS stimulation (47). LPS-stimulated *Irg1*^−^*^/^*^−^ BMDMs have significantly higher levels of fumarate and malate than WT controls, while succinate levels are almost that of unstimulated cells. The authors suggest that this is due to the ability of itaconate to inhibit SDH, and they showed itaconate to inhibit a purified form of SDH. In contrast to the decrease in oxygen consumption rate (OCR) seen in WT BMDMs upon treatment with LPS, in *Irg1*^−^*^/^*^−^ BMDMs OCR increases. As SDH also acts as complex II of the ETC, this highlights the ability of endogenous itaconate to regulate mitochondrial metabolism and is consistent with other reports of itaconate competitively inhibiting SDH, albeit weakly, the first of which was in 1949 (103–105). This led Lampropoulou et al. to suggest that LPS-induced *Irg1* expression and corresponding increase in intracellular itaconate is responsible for the second break in the Krebs cycle at SDH. By inhibiting SDH itaconate would also prevent the generation of ROS through RET (106). When succinate accumulates and is oxidized by SDH, it will produce a large amount of coenzyme Q. Electrons are then forced back through complex I of the ETC-generating ROS. ROS is able to activate the inflammasome and, therefore, drive the production of IL1β and IL18 (107).

IRG1 has also been shown to play a role in the establishment of endotoxin tolerance in LPS-tolerized macrophages. siRNA-mediated knockdown of IRG1 in these macrophages was able to increase NF-κB and IRF3 activation, while the production of ROS and subsequent expression of the zinc-finger protein A20 were reduced (90). Heme oxygenase 1 and carbon monoxide were able to induce IRG1 and through IRG1 downregulated pro-inflammatory gene expression in LPS-treated RAW264.7 cells and in a LPS mouse sepsis model (108). Several other elements regulating IRG1 expression and, therefore, itaconate production have also recently been identified. Computational modeling coupled with siRNA knockdown identified interferon regulatory factor 1 as a regulator of IRG1 transcription in RAW264.7 macrophages and human PBMCs (94). Inhibition of branched-chain aminotransferase 1 in human monocyte-derived macrophages decreased levels of glycolysis and oxygen consumption while also reduced IRG1 mRNA and protein levels as well as itaconate production (109).

A major issue with the study of the functional effect of itaconate in macrophages to date has been the use of DMI. DMI was utilized as it is cell permeable, however, it has been shown that while DMI boosts the level of itaconate in the cell it is not itself metabolized to itaconate (110). This suggests that DMI somehow promotes LPS-driven synthesis of itaconate. El Azzouny et al. also confirm that LPS-stimulated macrophages have increased succinate levels and that this is not the result of itaconate being metabolized to succinate. The authors speculate that the effects of DMI on macrophage metabolism may be due to an ability to act as a cysteine alkylating agent or to alter redox homeostasis. They further suggest that, though one has not been identified, it is possible a cell surface receptor for itaconate exists that DMI would be able to bind. Other metabolites have been shown to signal through G-protein-coupled receptors, such as succinate through GPR91, which has been renamed SUCNR1 (111). While the effects of studies carried out utilizing DMI have been drawn into question, the body of work carried out using genetic inhibition or deletion of *Irg1* and the striking amount by which *Irg1* mRNA and itaconate synthesis are upregulated in activated immune cells still leaves it worthy of further investigation.

## Conclusion

Our understanding of immune cell metabolism has come far since the early observations that activated macrophages were highly glycolytic (112, 113). It is now well accepted that these pathways play a part outside of their traditional energetic and biosynthetic roles. The discovery that the Krebs cycle is not complete in activated M1 macrophages and DCs highlights the importance of the withdrawal of citrate from the cycle for DC activation, the production of pro-inflammatory mediators and for the generation of itaconate. Citrate links many important cellular processes, bridging carbohydrate and fatty acid metabolism and protein modification. Its role in producing acetyl-CoA for the acetylation of histones may turn out to be its most striking role in regulating immune cell function. There is still much to be discovered regarding the regulation and consequences of metabolic reprogramming in immune cells, however, it is clear that a “citrate pathway” plays an important role in these processes and may be amenable to therapeutic targeting.

## Author Contributions

NW wrote the manuscript. LO’N supervised and edited the manuscript.

## Conflict of Interest Statement

The authors declare that the research was conducted in the absence of any commercial or financial relationships that could be construed as a potential conflict of interest.

## Abbreviations

ACC, acetyl-CoA carboxylase; ACLY, ATP-citrate lyase; ACO2, aconitase 2; ACSS, acetyl-CoA synthase; aKG, α-ketoglutarate; AMPK, AMP-activated protein kinase; ATP, adenosine triphosphate; BMDM, bone marrow-derived macrophage; CIC, citrate carrier; CPT1, carnitine palmitoyltransferase 1; CS, citrate synthase; DC, dendritic cell; DMI, dimethyl itaconate; ETC, electron transport chain; FAO, fatty acid oxidation; FASN, fatty acid synthase; FIH, factor inhibiting HIF; GAPDH, glyceraldehyde 3-phosphate dehydrogenase; HIF, hypoxia-inducible factor; HK2, hexokinase 2; ICL, isocitrate lyase; IDH, isocitrate dehydrogenase; IFN-γ, interferon-γ; IKKε, IκB kinase ε; iNOS, inducible nitric oxide synthase; IRF, interferon regulatory factor; IRG1, immune-responsive gene 1; LDHA, lactate dehydrogenase A; LPS, lipopolysaccharide; MDH, malate dehydrogenase; MPC, mitochondrial pyruvate carrier; MTB, *Mycobacterium tuberculosis*; mTOR, mammalian target of rapamycin; NAA, N-acetylaspartic acid; NF-κB, nuclear factor-κB; NK cell, natural killer cell; NO, nitric oxide; OCR, oxygen consumption rate; OXPHOS, oxidative phosphorylation; PDH, pyruvate dehydrogenase; PDK1, pyruvate dehydrogenase kinase 1; PFK, phosphofructokinase; PGE_2_, prostaglandin E_2_; PI3K, phosphatidylinositide-3-kinase; PK, pyruvate kinase; PPP, pentose phosphate pathway; RET, reverse electron transport; ROS, reactive oxygen species; SDH, succinate dehydrogenase; SREBP, sterol regulatory element-binding protein; TBK1, TANK-binding kinase 1; TLR, toll-like receptor; TNFα, tumour necrosis factor α.
